# Data from collection and analysis of RNA sequencing data from pearl millet

**DOI:** 10.1016/j.dib.2024.110592

**Published:** 2024-06-04

**Authors:** Kota Kambara, Shashi Kumar Gupta, Tetsuo Takano, Daisuke Tsugama

**Affiliations:** a Asian Research Center for Bioresource and Environmental Sciences (ARC-BRES), Graduate School of Agricultural and Life Sciences, The University of Tokyo, 1-1-1 Midori-cho, Nishi-tokyo-shi, Tokyo 188-0002, Japan; b International Crops Research Institute for Semi-Arid Tropics (ICRISAT), Patancheru, Telangana 502324, India

**Keywords:** Pearl millet, RNA sequencing, Transcriptome, Abiotic stress, gene

## Abstract

Pearl millet (*Pennisetum glaucum*, also known as *Cenchrus americanus*) is a cereal crop that has a C4 photosynthesis system and that can grow and develop seeds even under stressed conditions including drought-stressed, high temperature-stressed and nutrient-poor conditions. In previous studies, transcriptomes of pearl millet were studied by RNA sequencing (RNA-Seq) to understand mechanisms regulating its development and tolerance to such stressed conditions. Here, RNA-Seq reads from 565 pearl millet samples from 25 projects in the NCBI (National Center for Biotechnology Information) BioProject database were collected and mapped to the pearl millet reference genome to obtain read counts and transcripts per million (TPM) for each pearl millet gene. The count and TPM data for all the 565 samples as well as the attributes of those samples and projects were deposited in the figshare repository (https://doi.org/10.6084/m9.figshare.24902100).

Specifications Table

**Table:** 

Subject	Plant Science
Specific subject area	RNA present in various samples from pearl millet (*Pennisetum glaucum*)
Data format	Analyzed
Type of data	Table
Data collection	The NCBI (National Center for Biotechnology Information) BioProject database was searched for studies for RNA sequencing (RNA-Seq) of pearl millet samples with the keyword “pearl millet RNA-Seq” and “*Cenchrus americanus* RNA-Seq”, and this identified 25 projects as such. RNA-Seq reads and attributes from 565 samples from these 25 projects were downloaded. These reads were mapped to the pearl millet reference genome. Read counts and transcripts per million (TPM) for each pearl millet gene were obtained.
Data source location	NCBI BioProject data with the accession numbers indicated in the “BioProject” column in the “sample_attributes.xlsx” file: https://figshare.com/articles/dataset/Collection_of_pearl_millet_RNA_sequencing_data/24902100?file=43824531 (in the figshare repository indicated below)
Data accessibility	Repository name: figshare Direct URL to data: https://doi.org/10.6084/m9.figshare.24902100

## 1 Value of the Data

• These data can be used to compare expression levels of pearl millet genes between tissues, developmental stages and growth conditions.
• These data can help identify biological processes relevant to those tissues, stages and conditions.
• Pearl millet researchers and breeders will benefit from these data.
• Researchers and breeders for other crops can also benefit from these data.

## 2 Background

Pearl millet (*Pennisetum glaucum*, also known as *Cenchrus americanus*) is a cereal crop that has a C4 photosynthesis system and that is tolerant to stressed conditions such as drought-stressed, high temperature-stressed and nutrient-poor conditions. Pearl millet is a diploid with an approximately 1.79-Gb genome. The release of the reference genome of pearl millet enabled pearl millet genetics and genomics to be more accessible [1]. In previous studies, transcriptomes from various pearl millet samples were analyzed by RNA sequencing (RNA-Seq) to identify genes and biological processes regulating the pearl millet development and the tolerance to the stressed conditions [2,3, for example]. However, no comprehensive collection or database of analyzed RNA-Seq data, which can help to browse expression of genes in a wide range of samples, for pearl millet is available thus far (Table 1).

**Table 1:** Data deposited in the figshare repository.

File	Description
count_matrix_Pg.tsv	Tab-separated values of the read counts for all the pearl millet genes and the 565 pearl millet samples for RNA-Seq
count_tpm_Pg.tsv	Tab-separated values of the TPM for all the pearl millet genes and the 565 pearl millet samples for RNA-Seq
sample_attributes.xlsx	Attributes of the 565 samples in the Microsoft (MS) Excel format
sample_attributes.txt	Attributes of the 565 samples in a plain text format (with tab-separated values)
notes_pca_script.txt	Information about the above files and browsing them on pcaExplorer [5] on R
PM_RNA-seq_DB_script.txt	Scripts used for the RNA-Seq data analysis

## 3 Data Description

**Fig. 1:**
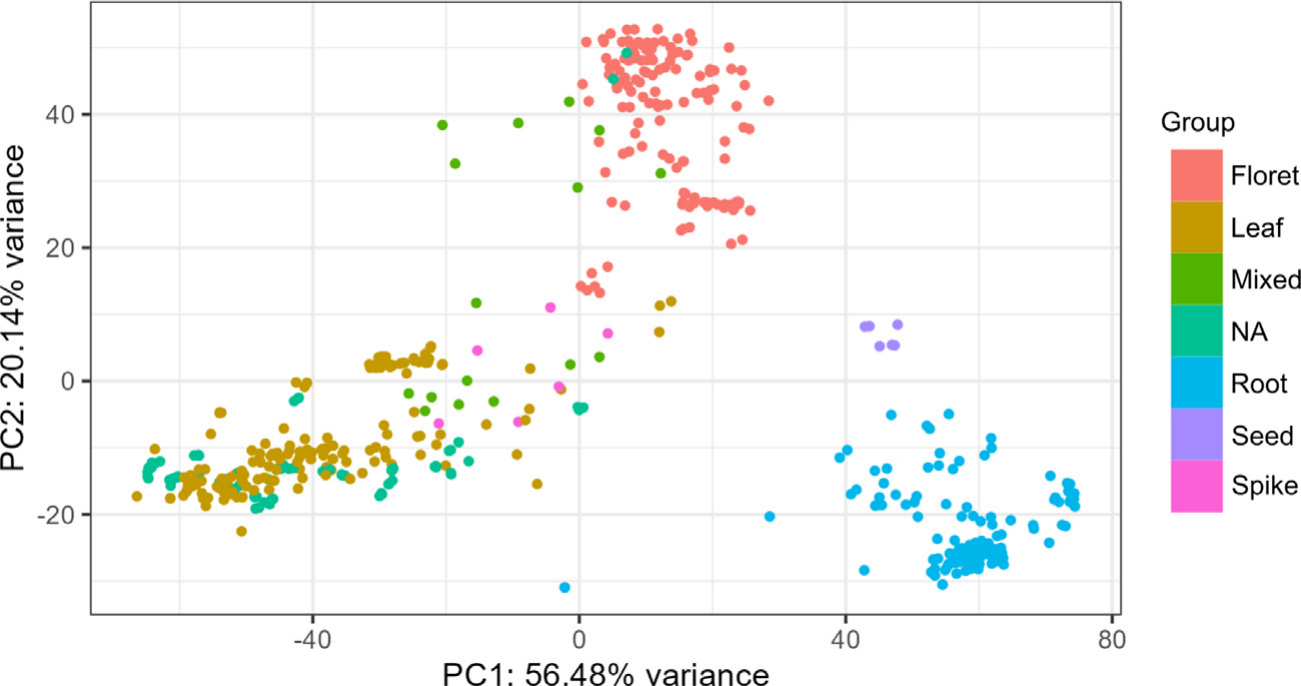
A plot of PC1 and PC2 from PCA with the 565 pearl millet samples for RNA-Seq. The plot was generated on pcaExplorer on R with the read count data for all the pearl millet genes and the sample attributes as the input. The group presented in the right side is based on the “tissue_modified” data in the sample attributes.

**Fig. 2:**
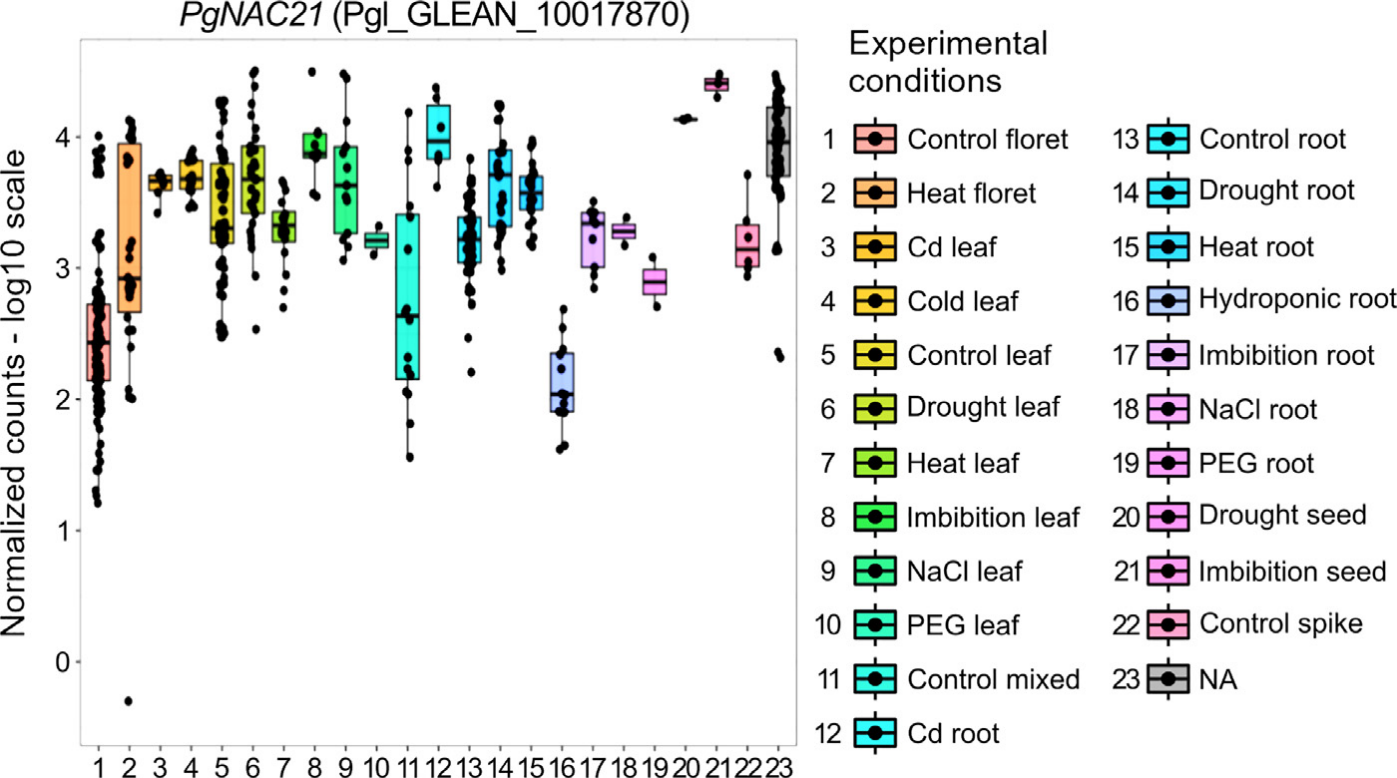
Normalized read counts for *PgNAC21* (Pgl_GLEAN_10017870). The plot was generated on pcaExplorer on R with the read count data and the sample attributes as the input. The group presented in the right side is based on the “treatment_modified” and “tissue_modified” data in the sample attributes.

RNA-Seq reads as well as attributes from 565 samples from 25 projects in the NCBI (National Center for Biotechnology Information) BioProject database were downloaded. These reads were mapped to the reference genome of pearl millet. Read counts and transcripts per million (TPM) for each pearl millet gene were obtained as gene expression values. Tables containing those attributes, read counts and TPM were deposited in the figshare repository (10.6084/m9.figshare.24902100). Principal components 1 and 2 (PC1 and PC2, respectively) derived from principal component analysis (PCA) with the above 565 samples are presented in Fig. 1. Normalized read counts of *PgNAC21*, a pearl millet gene involved in regulating responses to salinity stress [4], are presented in Fig. 2 as an example of the use of the data.

## 4 Experimental Design, Materials and Methods

The NCBI BioProject database was searched with either “pearl millet RNA-Seq” or “*Cenchrus americanus* RNA-Seq” as the keyword for the projects for RNA-Seq of pearl millet samples. RNA-Seq reads for the samples used for the resulting projects were downloaded by fasterq-dump (https://www.ncbi.nlm.nih.gov/sra/docs/toolkitsoft/) using their accession numbers for the NCBI sequence read archive (SRA) database (https://www.ncbi.nlm.nih.gov/sra), and mapped to the reference genome of pearl millet [1] by HISAT2 with default settings [6]. Read counts for each gene were obtained by featureCounts [7]. TPM were obtained from these read counts by a custom Python script. For the read counts and TPM, the data from the 565 samples were combined into single tables in the tab-separated values (TSV) format by a custom Perl script. Attributes for those 565 samples were downloaded from the NCBI BioSample database (https://www.ncbi.nlm.nih.gov/biosample) and changed manually to the TSV format with one row for one sample. The attributes (or columns) “treatment_modified”, “time_point”, “tissue_modified” and “stage_modified” were manually added as simplified attributes for the convenience in sample grouping. The tables for the read counts for all the pearl millet genes and the sample attributes were used as the input for pcaExplorer [5], where normalization of the read counts and the PCA were performed. The plot of PC1 and PC2 was obtained in “Samples View” on pcaExplorer, and the plot of the normalized counts for *PgNAC21* (accession number: Pgl_GLEAN_10017870 in [1]; MK084913 in NCBI GenBank (https://www.ncbi.nlm.nih.gov/nuccore/1524840355/) [4]) was obtained in “Gene Finder”.

## Limitations

None.

## Ethics Statement

This work meets the ethical requirements for publication in Data in Brief. This work does not involve human subjects, animal experiments, or any data collected from social media platforms.

## CRediT authorship contribution statement

**Kota Kambara:** Investigation, Data curation, Visualization, Writing – original draft. **Shashi Kumar Gupta:** Investigation, Data curation, Writing – original draft. **Tetsuo Takano:** Supervision, Conceptualization, Writing – review & editing. **Daisuke Tsugama:** Investigation, Data curation, Supervision, Conceptualization, Writing – original draft.

## Data Availability

Collection of pearl millet RNA sequencing data (Original data) (figshare).
